# Ethics and Jurisprudence

**Published:** 1915-05

**Authors:** 


ETHICS AND JURISPRUDENCE.—This is a new book from the pen of Dr. Edward Noyes, Professor of Ethics and Jurisprudence in Northwestern University Dental School. Any one who has kept in touch with dental literature of the middle west for the past quarter of a century will be familiar with the writings of Dr. Noyes, as he has been a frequent contributor to our periodic literature. Any one who knows the man will also know that whatever he may write on any subject will be pervaded with a spirit of honesty and fairness that commands one's confidence. The book before us is the expression of the spirit and controlling factors in a most loyal, professional man and devoted teacher. His ethics are placed on the highest plane of moral conception. He has endeavored to put into his teachings the high moral ideals that should predominate every professional man's life. It is difficult to review such a book, as there is so much that one wants to direct attention to that it would be like writing an essay to do any sort of justice to the subject. It is a book that ought to be read by every young dentist and it is also a book that will be most highly appreciated by the practitioner of years who has had to work out many of these problems in the hard methods of personal experience. If the spirit of the text of this volume could be transplanted into the activities of our profession we should soon realize that we are a profession in fact as well as in name, and that we are called to as exalted a service as any other cultured profession.

The chapters on jurisprudence are well stated and give one a good idea of his privileges and duties to his patients and the community in which he practices. Fortunately our profession has had no serious conflicts with the law. We have up to this time lived for the most part in a freedom that unconsciously has kept us out of harm from the laws. Our laws are, however, becoming more mandatory and somewhat complex and we should know our relations. For these and many other reasons this book should be in every dentist's library. It is published by the author and may be had through the supply houses.

